# Fatty Acid and Antioxidant Profile of Eggs from Pasture-Raised Hens Fed a Corn- and Soy-Free Diet and Supplemented with Grass-Fed Beef Suet and Liver

**DOI:** 10.3390/foods11213404

**Published:** 2022-10-28

**Authors:** Selin Sergin, Vijayashree Jambunathan, Esha Garg, Jason E. Rowntree, Jenifer I. Fenton

**Affiliations:** 1 Department of Food Science and Human Nutrition, Michigan State University, 469 Wilson Rd, East Lansing, MI 48824, USA; 2 Department of Animal Science, Michigan State University, 474 S Shaw Ln, East Lansing, MI 48824, USA

**Keywords:** poultry, yolk, omega-3, conjugated linoleic acid, GC-MS, branched-chain

## Abstract

There is increasing interest in using grass-fed beef (GFB) by-products to augment the nutrient profile of eggs among local pasture-raising systems in the US. The objective of this study was to characterize egg yolk fatty acid and antioxidant profiles using eggs from pasture-raised hens fed a corn- and soy-free diet and supplemented with GFB suet and liver compared to eggs from pasture-raised hens fed a corn and soy layer hen feed and commercially obtained cage-free eggs. The egg yolk vitamin and mineral profile was also assessed by a commercial laboratory. Both pasture-raised groups had twice as much carotenoid content, three times as much omega-3 fatty acid content, and a 5–10 times lower omega-6:omega-3 fatty acid ratio compared to the cage-free eggs (*p* < 0.001). Eggs from hens fed a corn- and soy-free feed and GFB by-products had half as much omega-6 fatty acid content and five times more conjugated linoleic acid, three times more odd-chain fatty acid, and 6–70 times more branched-chain fatty acid content (*p* < 0.001). Feeding pasture-raised hens GFB suet and liver reduces agricultural waste while producing improved egg products for consumers, but further research is needed to quantify optimal supplementation levels and the efficacy of corn- and soy-free diets.

## 1. Introduction

Poultry farmers in the United States (US) are increasingly utilizing pasture systems to raise and feed hens for egg production [1]. Pasture-raising systems are distinct from “free-range” production systems in that producers must provide pasture for consumption, though these designations are sometimes used interchangeably [2]. Pasture-raised hens are able to forage on phytochemically diverse grasslands and scavenge for invertebrates, allowing them access to nutrients such as fats, vitamins and minerals, and secondary compounds including antioxidants that benefit both animal and human health [3]. Pasture-raised egg systems contribute to regenerative agriculture systems which aim to improve human and ecosystem health, farm production, and food system resiliency by linking soil and livestock management [4]. Pasture and hens play mutually beneficial roles: planting diverse forages supports good soil quality, while hens can help control insects and weeds without pesticides and spread manure that fertilizes the soil. In contrast, within both caged and cage-free production systems, hens are not provided outdoor access and are fed a typical layer hen diet consisting mainly of corn and soy, high in omega-6 (*n*-6) fatty acids and low in omega-3 (*n*-3) fatty acids [2,5,6,7].

Pasture-raised egg systems not only benefit the environment but also typically produce eggs that have a more favorable nutrient profile for human health [1,8,9,10]. Eggs are a widely available source of proteins, fat, vitamins, and minerals [11]. Notably, the bioavailability of carotenoids is greater in eggs compared to other sources such as green leafy vegetables, likely due to the lipid content of egg yolks [11,12]. Further, egg yolks from pasture-raised hens typically have a healthier fatty acid and nutrient profile compared to egg yolks from caged hens [10]. A study comparing egg yolk *n*-3 fatty acids from pasture-raised to caged hens found that yolks from pasture-raised hens had 4.5 times more alpha-linolenic acid (ALA) and 2 times more eicosapentaenoic acid (EPA) and docosahexaenoic acid (DHA) than yolks from caged hens [1]. Providing pasture to hens yields egg yolks with a lower omega-6:omega-3 (*n*-6:*n*-3) ratio and significantly greater vitamin A, vitamin E, and carotenoid content compared to egg yolks from caged hens [1,9,10]. Further, egg yolks from pasture-raised hens contain significantly greater branched-chain fatty acids (BCFAs) compared to yolks from commercial free-range and caged eggs [10]. 

Livestock serves an important role in reducing food waste by converting inedible foods into high-value products, yet by-products of the livestock industry including fat and internal organs contribute to the accumulation of waste, economic losses, and environmental pollution [13,14]. About 27.5% of animal weight is wasted in beef slaughter [14]. While previous studies have examined the impact of both vegetable and animal by-product supplementation for layer hens on the egg nutrient profile, there is increasing interest in using by-products of grass-fed beef (GFB) production to augment the nutrient profile of eggs among local pasture-raising systems in the US [15,16,17,18,19,20]. By feeding hens otherwise under-utilized GFB suet and liver, pasture-raised egg and GFB systems can work in tandem to reduce the environmental impact of beef production and produce eggs that may be more beneficial for human health given that modifying the composition of hen diets can change the fatty acid, vitamin, and mineral content of eggs [11]. Beef fat is about 19% stearic acid, a saturated fatty acid (SFA) known to have a neutral effect on human LDL cholesterol [21,22]. Further, grass-fed cattle have consumed only grass or other forages prior to slaughter [23]. Accordingly, GFB generally has a lower *n*-6:*n*-3 ratio and higher conjugated linoleic acid (CLA) content compared to grain-fed beef [24]. GFB liver contains 92 mg ALA, 151 mg EPA, and 83 mg DHA per 100 g of liver; these fatty acid levels are significantly higher than those found in grain-fed cattle liver [25]. Additionally, GFB liver is a rich source of vitamin A, iron, zinc, and vitamin B12 [26]. While it should be noted that this supplementation practice may not reflect what is considered acceptable to feed layer hens by regulatory bodies in other countries, supplementing hen diets with these by-products among small-scale farms in the US may increase the deposition of these nutrients in eggs and thus confer health benefits to consumers. 

The objective of this study was to characterize egg yolk fatty acid and antioxidant profiles using eggs from pasture-raised hens fed a corn- and soy-free diet and supplemented with GFB suet and liver in comparison to eggs from pasture-raised hens fed a standard corn and soy layer hen feed and commercially obtained cage-free eggs. In addition, egg yolk vitamin and mineral profiles were assessed.

## 2. Materials and Methods

### 2.1. Chemicals

The gas chromatography–mass spectrometry (GC-MS) reference standard curve contained a mixture of Supelco 37 Component FAME Mix (Sigma-Aldrich, St. Louis, MO, USA) with mead acid, docosatetraenoic acid, *n*-3 docosapentaenoic acid (DPA), *n*-6 DPA, and palmitelaidic acid (Cayman Chemical, Ann Arbor, MI, USA). BCFAs were compared to Mixture BR 3 (Larodan AB, Solna, Sweden), while CLA isomers were compared to CLA reference standard UC-59M (Nu-Chek Prep, Elysian, MN, USA). Dichloromethane was purchased from VWR Chemicals (Radnor, PA, USA). All other chemicals were purchased from Sigma-Aldrich (St. Louis, MO, USA) unless otherwise noted.

### 2.2. Diet Characteristics and Collection

Pasture-raised, corn- and soy-free feed with beef by-products (PBB): Eggs were collected from hens on a farm in Southwest MI where hens were mobile pasture-raised in a 180-ft enclosed circle rotated every 3–4 days to move the hens to fresh pasture. The flock consisted of approximately 50 hens that were a mix of 1-year-old ISA Brown and Barred Rock hens. Hens were fed a custom feed of peas, barley, alfalfa, and calcium. Exact details regarding the composition of the custom layer hen feed cannot be shared to protect the farm’s custom recipe. However, feed samples were provided for fatty acid and antioxidant analysis. Hens were supplemented with GFB suet, GFB liver, and grit (crushed stone to support hen digestion) twice a week. The GFB suet and liver were obtained from a local farm that rotationally grazed cattle on a complex pasture mixture, moving cattle to fresh forage each day. The suet was obtained from fat around the internal organs of the cattle and was provided in raw, chopped pieces. Hens consumed about 30–60 g of liver per week. The suet was provided ad libitum in bins. Samples of the custom layer hen feed (*n* = 3) and the suet (*n* = 3) and liver (*n* = 3) were obtained from the farm. A dozen eggs were randomly collected on one day from the nest box, and from this, *n* = 6 eggs were randomly selected for analysis upon arrival to the laboratory. Layer hen feed samples were ground to pass a 1 mm screen in a Wiley mill (Arthur H. Thomas, Philadelphia, PA, USA) with dry ice and stored at −80 °C under nitrogen. The liver was prepared by mincing and finely grinding it in a crucible over dry ice, while the suet was finely minced before sampling. 

Pasture-raised, corn, and soy feed comparison (PCS): Eggs were collected for comparison from hens from a farm in Central-West MI where hens were managed similarly to the PBB group. Hens were mobile pasture-raised in a 180-ft enclosed circle rotated every 3–4 days to move the hens to fresh pasture. The flock consisted of approximately 50 hens that were a mix of 1-year-old ISA Brown and Barred Rock hens. Distinct from the PBB group, these hens were fed only a standard layer hen feed consisting of corn, soy, calcium, and grit obtained from a local feed mill in Michigan in addition to what they consumed from the pasture. Samples of the layer hen feed (*n* = 3) were obtained from the farm. A dozen eggs were randomly collected on one day from the nest box, and from this, *n* = 6 eggs were randomly selected for analysis upon arrival to the laboratory. Layer hen feed samples were ground to pass a 1 mm screen in a Wiley mill (Arthur H. Thomas, Philadelphia, PA, USA) with dry ice and stored at −80 °C under nitrogen.

Cage-free comparison (CFC): Cage-free eggs were collected for comparison from a local grocery store (*n* = 6 eggs). These eggs were labeled as “cage-free” and were from hens that would not have been provided pasture, according to USDA requirements for “cage-free” eggs [5]. It was not possible to obtain samples of the commercial layer hen diet.

### 2.3. Chemical Composition of Feeds

Chemical composition of the PBB and PCS layer hen feeds was assessed by Dairy One Forage Laboratory (Ithaca, NY, USA) using respective AOAC methods.

### 2.4. Physical Characteristics of Eggs

Physical characteristics were measured upon collection of the eggs as previously described [10]. Briefly, albumen, yolk, and shell weights were determined, and albumen height was measured using a micrometer. Haugh units were calculated using albumen height and egg weight (Haugh unit = 100 × log (albumen height + 7.57 − 1.7 × egg weight^0.37^) [27]. Egg yolk color was determined using the DSM Yolk Color Fan (DSM Nutritional Products, Basel, Switzerland) with values ranging from 1 for pale yellow to 14 for dark orange. After being analyzed for physical characteristics, egg yolks were immediately freeze-dried, ground, and stored at −80 °C under nitrogen.

### 2.5. Phenolic Analysis of Eggs and Feeds

Total phenolic content was assessed using spectrophotometric screening methods as previously described by Sergin et al. [10] using a modified extraction method based on Nimalaratne et al. [28] and the Folin–Ciocalteu assay modified from Chen et al. [29]. Briefly, two extractions, first with 20 mL of a methanol/distilled water/acetic acid solvent [80:18:2 (*v*/*v*/*v*)], and second with 20 mL of an acetone/distilled water/acetic acid solvent [80:18:2 (*v*/*v*/*v*)], were used to extract phenolic compounds from 2 g of lyophilized egg yolk sample or ground layer hen feed. Tubes were shaken and centrifuged (840× *g*, 4 °C) and supernatants were combined following the addition of each solvent as previously described [10]. Next, 100 μL Folin–Ciocalteu reagent and 800 μL 5% sodium bicarbonate were added to a gallic acid standard curve (1 mg/mL to 0.002 mg/mL) and to 100 μL of the supernatant. These samples were heated at 40 °C for 30 min, cooled at room temperature for 10 min, and plated in triplicate in a 96-well plate. Samples were then scanned in a microplate reader (Bio-Tek, Winooski, VT, USA) at 765 nm, compared against the standard curve, and reported as milligrams of gallic acid equivalents (GAE) per gram of fresh egg yolk or feed.

### 2.6. Egg Carotenoid Analysis

Egg yolk total carotenoid content was determined using spectrophotometric screening methods as described by Sergin et al. [10] using methods adapted from Chen et al. [29] and Rodriguez-Amaya and Kimura [30]. Briefly, 0.5 g of lyophilized egg yolk sample and 5 mL of cold acetone (0.05% butylated hydroxytoluene (BHT)) were combined and homogenized. Samples were vortexed for 2 min, placed in an ultrasound water bath for 5 min, and centrifuged for 15 min (1200× *g*, 4 °C). The supernatant was evaluated in a UV–Vis Double Beam Spectrophotometer (VWR, Radnor, PA, USA) at 450 nm against an acetone blank. Total carotenoid content was calculated according to Biehler et al. [31] using an ε of 140,663 L/mol for beta-carotene in acetone and was expressed as micrograms of beta-carotene per gram of fresh egg yolk.

### 2.7. Layer Hen Feed Carotenoid Analysis

Total carotenoids of the layer hen feeds were determined using spectrophotometric screening methods adapted from Lichtenthaler and Wellburn [32]. In a conical tube, 2 g of ground layer hen feed was combined with 20 mL of 70% aqueous acetone. The tubes were shaken for 30 min and centrifuged for 20 min at 840× *g* and 4 °C. The supernatant was recovered in a new tube. The extraction was repeated with an additional 20 mL of 70% aqueous acetone and the supernatants were pooled. Using the spectrophotometer, carotenoid and chlorophyll content of the supernatants were assessed in glass cuvettes at three wavelengths (663, 646, and 470 nm). Chlorophyll A, chlorophyll B, and total carotenoids were calculated using the following equations:

$$\mathrm{Chlorophyll}\;a\;\left(C_{a}\right)=12.21A_{663}-2.81A_{646}$$

$$\mathrm{Chlorophyll}\;b\;\left(C_{b}\right)=20.13A_{646}-5.03A_{663}$$

$$\mathrm{Total}\;\mathrm{carotenoids}=\frac{1000A_{470}-3.27C_{a}-104C_{b}}{229}$$

Total carotenoids were reported as micrograms per gram of feed.

### 2.8. Fatty Acid Analysis

The fatty acid profile of the lyophilized egg yolks, ground layer hen feed, and grass-fed beef suet and liver tissue was assessed as previously described by Sergin et al. [10]. Briefly, a modified version of the microwave-assisted extraction method by Bronkema et al. [33] was used to extract fatty acids using 400 mg of each sample and 8 mL of a 4:1 (*v*/*v*) ethyl acetate/methanol solution with 0.1% BHT as an antioxidant. Fatty acids were extracted in a CEM Mars 6 microwave (CEM Corp., Matthews, NC, USA) using the following microwave parameters: 55 °C for 15 min with an initial ramp of 2 min at 400 W maximum power. Samples were then filtered and prepared as previously described [10].

Methylation described by Sergin et al. [10] modified from Jenkins [34] was conducted to create fatty acid methyl esters (FAMEs). Two milligrams of extracted oil were combined with 500 µL toluene and 20 µg of methyl-12-tridecenoate (U-35M, Nu-Chek Prep, Elysian, MN, USA) as an internal standard. Base-catalyzed methylation was conducted using 2 mL of anhydrous potassium methoxide (0.5 N) at 50 °C for 10 min. Then, acid-catalyzed methylation was conducted using 3 mL of methanolic HCl (5%) at 80 °C for 10 min. Two milliliters of HPLC water were added, then FAMEs were extracted twice using 2 mL of hexane. Extracted FAMEs were resuspended in 1 mL of isooctane and stored at −20 °C until GC-MS analysis.

FAMEs in the samples were separated using the HP-88 column with a 100 m length, 0.25 mm inner diameter, and 0.2 µm film thickness (Agilent Technologies, Santa Clara, CA, USA) on a Perkin Elmer 680/600 GC-MS (Waltham, MA, USA) in the electron impact mode with helium as the carrier gas (1 mL/min). For improved separation of fatty acid isomers, column temperature parameters described by Kramer et al. [35] were used as follows: initial temperature of 80 °C for 4 min, ramp at a rate of 13.0 °C/min to 175 °C, held for 27 min, ramp at a rate of 4.0 °C/min to 215 °C, and held for 35 min. Two different injections with a 1 µL injection volume and 250 °C injection temperature were conducted to measure both lower- and higher-concentration analytes. These were a 30:1 split injection and a splitless injection (0.75 min splitless hold time, 40 mL/min flow exiting the vent). Regarding the MS, electron energy was 70 eV, and the transfer line and ion source temperature were set to 180 °C. MS data were recorded in full scan mode (*m/z* 70–400).

Data were analyzed using MassLynx (4.1 SCN 714; Waters Corp., Milford, MA, USA). Retention time and EI mass fragmentation of each analyte were compared to those in the reference standard (described above). Fatty acids not included in the reference standard were identified by elution order as reported by Kramer et al. [35] and confirmed with EI mass fragmentation. Fatty acids were quantified using extracted ion chromatograms of the corresponding quantitative ions utilizing a standard curve constructed from our reference and internal standard. To calculate each FAME concentration, the internal standard peak area and analyte peak area in each sample were compared to those of the standard curve. Fatty acids were reported as percent of total fatty acids quantified and in gram amounts based upon the total oil yield from the microwave-assisted extraction.

### 2.9. Egg Yolk Vitamin and Mineral Analysis

For the egg vitamin and mineral analysis, remaining lyophilized egg yolk powder was pooled to form three replicates per group consisting of two egg yolks each. Egg yolk vitamin and mineral analyses were conducted by a commercial laboratory (Creative Proteomics, Shirley, NY, USA). Vitamins were analyzed using targeted metabolite quantitation using LC-MRM/MS and minerals were quantified using ICP-MS.

### 2.10. Statistical Analysis 

Based on a power analysis conducted using changes in the *n*-6:*n*-3 ratio of egg yolks from Sergin et al. [10], *n* = 6 egg yolks was determined to be sufficient to detect significant differences. The egg characteristics, feed chemical composition, phenolic, and carotenoid data were analyzed using Prism Mac 7.0d (GraphPad Software, La Jolla, CA, USA). The fatty acid and vitamin and mineral data were analyzed using R version 3.6.1. Independent samples *t*-tests were performed for all the feed analyses. Group comparisons for all the egg analyses were performed using one-way analysis of variance (ANOVA) and Tukey’s HSD with correction for multiple comparisons. Values under the lower limit of detection were treated as zeroes. Statistical significance was set at *p* < 0.05 for all analyses.

## 3. Results and Discussion

### 3.1. Nutrient Composition of the Layer Hen Diets

The chemical composition and antioxidant profile of the PBB and PCS layer hen feeds are shown in Table 1.

The main fatty acid profile of the GFB liver, GFB suet, and layer hen feeds are reported in Table 2 and Table S1.

### 3.2. Egg Physical Characteristics and Antioxidant Profiles

Egg physical characteristics are presented in Table 3 and egg yolk antioxidant profiles are presented in Table 4.

Egg weight, yolk weight, shell weight, albumen weight, and Haugh units were not significantly different among the egg groups. Egg yolk color was significantly more orange in PBB and PCS eggs compared to CFC eggs. Carotenoids, which have important antioxidant properties, give egg yolks their orange–yellow color [11]. Accordingly, the screening analysis revealed that PBB and PCS egg yolks had significantly greater carotenoid content than CFC egg yolks (*p* < 0.001). Egg yolks from pasture-raised hens contained higher total carotenoids due to access to green vegetation, a source of a variety of carotenoids [1,9,10,36]. Current recommendations gathered from previous studies advise the consumption of at least 7 mg of beta-carotene per day to ensure adequate vitamin A intake [37]. According to this recommendation, consuming two PBB or PCS egg yolks provides approximately 20% of recommended intake, while two CFC egg yolks only provide about 8%.

In general, all three egg groups had a low total phenolic content, ranging from 0.60–1.80 mg GAE/g of fresh yolk as shown by the screening analysis (Table 4). Similar studies have observed a total phenolic content in eggs between 1.1–1.7 mg GAE/g of fresh yolk [10,38]. The higher phenolic content of the PCS and CFC egg yolks is likely due to phenolic compounds that are commonly found in cereal grains, including corn, which is a component of standard layer hen feeds [28]. Given that the deposition of phenolic compounds into egg yolks is low and that average polyphenol intakes of diets across varying dietary patterns are estimated to range from 300–1700 mg/day, egg consumption is unlikely to make a large contribution to polyphenol intake [28,39].

### 3.3. Egg Yolk Fatty Acid Profiles

The differences in the egg yolk fatty acid profile among the egg groups, shown in Table 5, can be partially attributed to differences in the hen diets. There was no difference in percent SFA among the egg groups, but there were significant differences in percent MUFA and PUFA. In general, the proportions of SFA, MUFA, and PUFA were consistent with other studies comparing egg yolks from hens fed conventional and pasture-containing diets, except for the low percent PUFA in PBB egg yolks [7,40]. An additional table displaying absolute quantification of the fatty acid profile in the egg yolks in grams of fatty acids per 100 g of fresh yolk is available in Table S2.

#### 3.3.1. Saturated Fatty Acids

Despite the inclusion of beef suet, high in SFAs, in the PBB diet, there was no difference in total SFAs by percentage or absolute quantification (Table 5 and Table S2). Only myristic acid was significantly higher in PBB egg yolks. These results are consistent with previous studies showing little to no differences in SFA concentration in hen egg yolks regardless of hen diet composition. One study compared the effects of the inclusion of seal blubber oil, high in *n*-3 PUFAs and MUFAs, to a control diet containing tallow, high in SFAs, on egg yolk fatty acid profile [15]. They observed SFA concentration ranging from 35–37% in all treatment groups, similar to the present study. The concentration of myristic acid was greater and stearic acid was lower in the egg yolks from hens fed 5% seal blubber oil compared to the egg yolks from hens fed 5% tallow. Despite these individual fatty acid differences, there were no observed differences in total SFA concentration among the egg yolks from hens fed different diets [15]. Another study compared the effects of fish oil, high in PUFAs, and tallow supplementation on the egg yolk fatty acid profile [16]. Egg yolk percent SFA ranged from 39–42%. The yolks from hens fed 100% of fat from tallow had significantly lower total SFA content than the yolks from hens fed 100% of fat from fish oil. Similarly, Celebi and Macit [17] and Grobas et al. [18] found that increasing the amount of tallow supplementation decreased the concentration of egg yolk SFAs. However, these differences, as stated by Baucells et al. [16], are negligible, demonstrating how the degree of saturation in egg yolks is maintained within very narrow margins [16]. 

#### 3.3.2. Mono- and Poly-Unsaturated Fatty Acids

Percent MUFAs were significantly higher in PBB eggs, while percent PUFAs were significantly higher in PCS and CFC eggs compared to PBB eggs (Table 5). This difference in percent total MUFAs was due to the lower gram amount of PUFAs in PBB eggs and not due to an actual increase in grams of MUFAs. Though the percent total MUFA and percent oleic acid were significantly greater in the PBB eggs compared to PCS and CFC eggs, there were no significant differences for grams of total MUFA or grams of oleic acid per 100 g of egg yolk (Table S2). This finding is consistent with previous studies demonstrating the limited ability of hens to increase egg yolk oleic acid content [16,41]. PBB eggs contained about half as many grams of total PUFA per 100 g of egg yolk compared to PCS and CFC eggs. This large difference was due to the significantly lower grams of linoleic acid (LA) in PBB egg yolks. Normally LA contributes >80% of total PUFA in egg yolks, seen in PCS and CFC eggs with about 81% and 90%, respectively [42]. However, LA constituted only 68% of total PUFA in the PBB eggs (Table S2). The corn- and soy-free PBB feed contained significantly fewer grams of total PUFA and LA compared to the corn- and soy-based PCS feed, which helps to explain the differences between the PBB and PCS egg yolks (Table S1).

The differences in *n*-6 PUFA and *n*-3 PUFA concentration among the egg groups further demonstrates the impact of the production system on egg yolk fatty acid profile. While the egg yolk ALA concentration was significantly higher in PCS eggs compared to CFC eggs, the egg yolk DPA and DHA concentrations were significantly higher in both PBB and PCS eggs compared to CFC eggs. Hens convert ALA obtained from their diet into these long-chain *n*-3 PUFAs via liver elongases and desaturases. The same enzymes can convert LA into arachidonic acid (C20:4 *n*-6) [43]. Though LA concentration was significantly lower in PBB eggs, there was no difference in arachidonic acid concentration compared to the other groups. PUFA metabolism, including whether the conversion of ALA or LA is favored, is affected by diet, genotype, age, and rearing system [43]. The hen’s access to forages in the PBB and PCS groups provided greater access to *n*-3 precursors than would have been available in the CFC-rearing system [44]. This difference likely contributed to greater conversion of ALA into DPA and DHA in the PBB and PCS egg groups. It should be noted that EPA was below the lower limit of detection in all the egg groups, indicating that EPA was converted into the longer chain *n*-3 PUFAs detected in the PBB and PCS groups. Numerous studies have found egg yolk EPA concentration to be below the limit of detection when feeding standard layer hen diets, and that supplementation with additional exogenous sources of ALA or EPA from supplements such as flaxseed or fish oil is necessary to increase egg yolk EPA concentration [45]. Though the GFB liver provided about 60 mg of EPA per 100 g to the PBB group, this was not sufficient to increase egg yolk EPA.

Further, percent *n*-6 PUFAs were lowest in PBB eggs, while percent *n*-3 PUFAs were significantly greater in the PBB and PCS eggs compared to CFC eggs. Thus, the *n*-6:*n*-3 ratios were significantly different among the egg groups, with a 51:1 ratio observed in CFC eggs compared to 6:1 and 11:1 in PBB and PCS eggs, respectively, from hens with unlimited access to fresh pasture (*p* < 0.001) (Table 5). This is congruent with previous studies showing that pasture access increases *n*-3 PUFA concentration and decreases the *n*-6:*n*-3 ratio of hen egg yolks [1,10,40,46]. Consuming diets with a lower *n*-6:*n*-3 ratio, around 4:1, is associated with a decreased risk of many chronic diseases including obesity and cardiovascular disease [47]. Thus, PBB and PCS egg yolks, compared to CFC egg yolks, are more aligned with nutrition recommendations to reduce *n*-6 PUFA and increase *n*-3 PUFA consumption [48,49].

#### 3.3.3. Conjugated Linoleic Acid

Conjugated linoleic acid is reported to possess potential anti-atherogenic and anti-obesogenic properties, have cancer-protective effects and modify immunity by reducing pro-inflammatory cytokines, though the health effects of CLA are still under investigation [50,51]. Percent conjugated linoleic acid in the beef liver and suet, layer hen feeds, and egg yolks are displayed in Table 6. Beef liver and suet were sources of CLA in the PBB hen diet. Thus, PBB eggs had a significantly higher CLA concentration compared to PCS and CFC eggs from hens who consumed corn and soy feed. A previous study comparing pasture-raised, free-range, and conventional egg yolks found no differences in CLA concentration by production system [10]. Similarly, there was no difference in CLA concentration between the pasture-raised PCS eggs and the conventionally raised CFC eggs. Thus, it is likely that the increased CLA in the PBB eggs was due to the hens’ animal-product consumption. Similarly, Shinn, et al. [52] demonstrated that feeding CLA-rich soy oil to hens increased the CLA concentration in egg yolks. CLA-rich oil appears to be more effective at increasing egg yolk CLA compared to adding animal products to hen diets, achieving 2% CLA compared to 0.3% CLA in PBB egg yolks in the present study [52]. However, adding CLA-rich oil to hen diets also undesirably increased egg yolk SFA content and decreased MUFA content [52,53]. This effect was not seen with the addition of CLA from beef by-products in this study. Despite the beneficial effect of beef by-product supplementation on egg yolk CLA concentration, the effects on human health may be negligible. To yield health benefits, the CLA content of PBB eggs is relatively low with about 0.05 g of CLA per 100 g of fresh yolk (Table S2). Previous studies have estimated that consumption of 620 and 441 mg of CLA/day for men and women, respectively, was necessary to achieve a cancer-protective effect, and at least 3 g of CLA/day was needed to achieve body fat reduction [54,55].

#### 3.3.4. Odd-Chain Fatty Acids

Generally, OCFAs constitute a small amount of total fatty acids in foods; milk, which is one of the main dietary sources of OCFAs, contains 1% C15:0 and 0.5% C17:0 [56]. In contrast to even-chain saturated fatty acids, dietary intake of odd-chain saturated fatty acids is associated with a lower risk of inflammation, cardiovascular disease, type 2 diabetes, and other chronic diseases according to several human epidemiological studies [56,57,58,59,60]. Intake of OCFAs was also associated with lower total mortality in both males and females in a 14-year prospective cohort study with 14,000 participants [61]. Venn-Watson, et al. [56] demonstrated that OCFAs have biological effects that align with these health benefits in vitro and in vivo in animal studies. The substitution of 0.1% of energy or about 220 mg of PUFAs or MUFAs in a 2000 kilocalorie diet with 220 mg of OCFAs was associated with 15–19% lower total mortality in men and women [61].

Odd-chain fatty acid concentration is not frequently reported in eggs. Limited evidence indicates egg yolk OCFA concentration ranges from 0.24–0.43%, with no significant differences observed among the production systems studied, similar to the OCFA concentration in PCS and CFC eggs in this study (Table 6) [10,62]. However, PBB eggs contained 0.95% OCFAs (Table 6). Further, per yolk, PBB eggs contained significantly more OCFAs than PCS or CFC eggs (0.15 g vs. 0.06 g or 0.04 g per 100 g of fresh yolk: Table S2). The beef liver, beef suet, and PBB feed were all sources of OCFAs in the PBB hen diet, and the PBB layer hen feed had a significantly higher concentration of OCFAs than the PCS feed (Table 6). This suggests that the inclusion of beef by-products and the PBB feed contributed to increasing the OCFA concentration in PBB egg yolks rather than pasture access. Interestingly, Evans and LaMont [63] demonstrated that the inclusion of microalgae to create an OCFA-enriched layer hen feed can increase egg yolk OCFAs to at least 3% of total fatty acids. Effective methods to increase the OCFA content of hen eggs need to be further studied to help consumers increase their OCFA intake and achieve the observed health benefits of OFCA consumption.

#### 3.3.5. Branched-Chain Fatty Acids

To our knowledge, this is one of the few studies to characterize BCFA isomers in egg yolks. BCFAs are found primarily in animal products such as beef and dairy, at around 1.89% and 2.05% of total fatty acids, respectively [64]. Health information about BCFAs is limited, but they are thought to function similarly to *cis*-unsaturated fatty acids due to their branched structure [64]. BCFAs reduced the incidence of necrotizing enterocolitis and increased the expression of anti-inflammatory cytokines in a rat model, demonstrated anticancer properties in vitro, and modulated gut microbiota composition in vivo [65,66,67,68].

PBB eggs contained greater amounts of all BCFA isomers compared to PCS and CFC eggs (Table 6 and Table S2). BCFAs were not detected in either the PBB or PCS feed, but both the GFB suet and liver were sources of BCFA isomers (Table 6). A previous study characterizing egg yolk BCFA isomers found that pasture-raised eggs had a higher BCFA concentration than free-range and commercially raised eggs, hypothesizing that the higher BCFA concentration may be related to differences in pasture access and microbe deposition from the hens’ microbiota, containing BCFAs, during egg formation [10]. However, egg yolks from pasture-raised hens in that study only contained about 0.05% BCFAs [10]. PBB egg yolks from hens with both pasture access and animal product consumption contained 0.59% BCFAs, around ten times that of the previous study. This suggests that feeding PBB hens animal products increased the BCFA content of the egg yolks in this study. PBB eggs contained about 0.09 g of BCFAs per 100 g of fresh yolk, a small portion of the typical daily BCFA intake in Western diets of around 0.4–0.5 g (Table S2) [64,67].

### 3.4. Egg Yolk Vitamin and Mineral Profiles

The vitamin profile of the egg yolks is shown in Table 7. Due to the high lipid content of egg yolks, lipid-soluble vitamins are easily incorporated into egg yolks [11]. There were no significant differences in egg yolk vitamin A, D3, or E content in this study. Previously, pasture access significantly increased the vitamin A and E content of hen egg yolks [1,9,10]. Similar to the present study, Anderson [69] found that free-range and caged hens had similar vitamin A content, while carotenoids were higher in free-range eggs. However, it is surprising that PBB eggs did not contain more vitamin A given that beef liver is a significant source of vitamin A, providing roughly 2000 retinol activity equivalents per ounce [70]. Since vitamin A is a recommended component of layer hen feeds, it is possible that the layer hen diets provided to the PCS and CFC groups provided a similar vitamin A content to that in the PBB diet [71]. The lack of differences in vitamin E content in this study may be due to the assessment of free alpha-tocopherol rather than total alpha-tocopherol which is typically reported in eggs [72,73]. Eggs are one of the few food sources rich in vitamin D due to the efficient transfer of vitamin D from hen diets into egg yolks and increasing hen dietary concentrations of vitamin D increases egg yolk vitamin D content accordingly [74]. The lack of differences in vitamin D3 content in the present study indicates there were similar vitamin D3 contents among PBB, PCS, and CFC diets. There were no differences in vitamin K2 content, while vitamin K1 was greater in PCS eggs compared to CFC eggs. Further, vitamin K1 was only detected in the pasture-raised egg groups. Access to green vegetation, a source of vitamin K1, may contribute to this difference [75].

The B-vitamin profile is not frequently reported in egg yolks. There were no differences in vitamin B2 or B9 content of the eggs (Table 7). Vitamin B1, found in higher amounts in corn and soy compared to beef liver, was greater in PCS eggs compared to PBB and CFC eggs [76]. The difference in B1 content between PCS and CFC eggs could be due to differences in the quality or preparation of the corn and soy feed provided in either diet [76]. Further, beef liver is a richer source of vitamin B3 compared to corn and soy, potentially explaining the higher B3 content of PBB eggs [76]. Regarding B5, corn and soy contain significantly less B5 than beef liver, yet PBB and PCS eggs had similar B5 content, nearly twice that of CFC eggs [76,77]. Given that vitamin B5 can be synthesized microbially, pasture access may have played a role in altering hen microbial communities and thus increasing vitamin B5 content [76,78]. Lastly, the B12 content was higher in CFC eggs compared to the other groups.

There were no significant differences in the mineral profile of the egg groups except for manganese content which was greater in the PCS eggs compared to the other groups (*p* < 0.001) (Table 8). The lack of differences in mineral content was surprising since beef liver is a rich source of iron, phosphorus, and zinc [79]. These minerals were expected to be greater in PBB eggs due to the inclusion of beef liver in the hen diets. Assessing a greater sample size, particularly for iron content, may be necessary to better elucidate any significant differences. 

## 4. Conclusions

This study is limited by the lack of specific layer hen diet composition information for PBB and PCS eggs and the inability to obtain diet information for CFC eggs. We were also unable to measure total phenolics and carotenoids using a more sensitive HPLC methodology. Further, this study was unable to connect egg yolk vitamin and mineral profiles to hen dietary factors. Since most previous egg studies mainly investigate tallow supplementation rather than suet and do not typically specify the source of the fat provided, it was difficult to make comparisons to the GFB suet provided in the present study. It is not clear whether understanding these distinctions would make a significant difference in this discussion. Lastly, since this study characterizes the nutrient profile of eggs from hens fed animal by-products in a small-scale farm in the US, it may not necessarily reflect what is allowed by regulatory bodies in other countries or in a commercial setting. This should be taken into account in future studies on this supplementation practice.

However, to our knowledge, this is the first study to report the nutrient profile of egg yolks from hens fed GFB suet and liver in a local, pasture-raised setting. In this study, both groups with unlimited access to fresh pasture had a higher egg yolk *n*-3 PUFA and carotenoid content and a lower *n*-6:*n*-3 ratio than commercially obtained eggs. Likely due to the corn- and soy-free diet, PBB eggs contained significantly less n-6 PUFAs than eggs from the other groups in this study. Moreover, feeding GFB suet and liver to the PBB group increased CLA, OCFA, and BCFA content without an increase in total SFA content but had a limited impact on the vitamin and mineral profile. These results demonstrate that providing GFB by-products and exploring alternative layer hen feeds have beneficial impacts on the egg nutrient profile that favor human health and reduce agricultural waste. Future studies should investigate differences in sources of beef tallow or suet and liver and the optimal quantity to achieve desired effects on the egg nutrient profile. Further investigation is needed to determine the efficacy of using alternatives to corn and soy in layer hen diets, particularly in pasture-raised systems.

## Figures and Tables

**Table 1:** Chemical composition and antioxidant profile of the layer hen feeds ^1^.

Parameters	PBB Feed	PCS Feed	CFC Feed	*p*-Value ^2^
Dry matter (%)	86.8	88.5	N/A	-
Crude protein (% DM)	17.2 ± 0.1	19.7 ± 0.2	N/A	<0.001
Metabolizable energy (Mcal/kg)	3.4 ± 0.0	3.5 ± 0.0	N/A	0.002
Total carotenoids (µg/g feed)	5.38 ± 0.48	7.20 ± 0.37	N/A	0.007
Total phenols (mg of GAE/g feed)	0.84 ± 0.05	1.06 ± 0.13	N/A	0.057

^1^ Means ± standard deviation (*n* = 3 per group). ^2^ Results of independent samples *t*-test between PBB feed and PCS feed. PBB feed, custom feed of peas, barley, alfalfa, and calcium; PCS feed, standard corn and soy feed; CFC feed, cage-free comparison; DM, dry matter, GAE, gallic acid equivalents; N/A, no data.

**Table 2:** Fatty acid profile of grass-fed beef by-products and layer hen feeds (% of total fatty acids) ^1^.

Fatty Acid	Beef Liver	Beef Suet	PBB Feed	PCS Feed	*p*-Value ^2^
10:0	<LLOD	0.05 ± 0.01	0.03 ± 0.00	0.02 ± 0.00	0.006
12:0	0.01 ± 0.00	0.07 ± 0.01	0.01 ± 0.00	0.01 ± 0.00	0.016
14:0	0.32 ± 0.05	3.89 ± 0.07	0.21 ± 0.01	0.06 ± 0.00	0.002
14:1	0.03 ± 0.01	0.29 ± 0.02	<LLOD	<LLOD	-
16:0	14.36 ± 0.28	26.33 ± 0.21	17.70 ± 0.47	12.01 ± 0.42	<0.001
16:1 *n*-7	0.50 ± 0.01	1.64 ± 0.09	0.12 ± 0.03	0.07 ± 0.01	0.120
16:1 *n*-7 *t*	0.21 ± 0.04	0.12 ± 0.02	<LLOD	<LLOD	-
16:1 *n*-9	0.25 ± 0.06	0.37 ± 0.06	0.06 ± 0.01	0.04 ± 0.01	0.135
18:0	35.61 ± 0.62	23.74 ± 0.58	2.14 ± 0.08	2.57 ± 0.06	0.003
18:1 *n*-7	0.84 ± 0.08	0.98 ± 0.07	0.65 ± 0.04	0.97 ± 0.11	0.023
18:1 *n*-9	8.08 ± 0.26	30.21 ± 0.68	19.43 ± 0.40	20.93 ± 0.08	0.020
18:1 *n*-9 *t*	1.53 ± 0.13	5.42 ± 0.35	<LLOD	<LLOD	-
18:2 *n*-6	4.11 ± 0.14	0.80 ± 0.04	51.10 ± 0.91	56.14 ± 0.40	0.004
18:3 *n*-3	2.18 ± 0.20	0.79 ± 0.10	7.06 ± 0.17	6.31 ± 0.61	0.162
18:3 *n*-6	0.26 ± 0.05	<LLOD	<LLOD	<LLOD	-
20:0	0.11 ± 0.03	0.14 ± 0.02	0.32 ± 0.02	0.21 ± 0.02	0.003
20:1 *n*-9	<LLOD	0.25 ± 0.01	0.42 ± 0.04	0.19 ± 0.02	0.003
20:2 *n*-6	0.24 ± 0.11	<LLOD	<LLOD	<LLOD	-
20:3 *n*-6	3.39 ± 0.16	<LLOD	<LLOD	<LLOD	-
20:4 *n*-6	7.12 ± 0.43	<LLOD	<LLOD	<LLOD	-
20:5 *n*-3	3.28 ± 0.12	<LLOD	<LLOD	<LLOD	-
22:0	<LLOD	0.07 ± 0.01	0.16 ± 0.02	0.18 ± 0.02	0.406
22:4 *n*-6	2.32 ± 0.40	<LLOD	<LLOD	<LLOD	-
22:5 *n*-3	9.83 ± 0.55	<LLOD	<LLOD	<LLOD	-
22:6 *n*-3	2.55 ± 0.05	<LLOD	<LLOD	<LLOD	-
24:0	<LLOD	<LLOD	0.31 ± 0.02	0.18 ± 0.03	0.003
Total SFA	51.90 ± 0.49	56.28 ± 0.35	21.16 ± 0.63	15.34 ± 0.39	<0.001
Total MUFA	11.68 ± 0.53	39.69 ± 0.21	20.68 ± 0.45	22.21 ± 0.09	0.023
Total PUFA	35.50 ± 0.40	1.85 ± 0.16	58.16 ± 0.74	62.45 ± 0.32	0.004
Total *n*-6	17.44 ± 0.28	0.80 ± 0.04	51.10 ± 0.91	56.14 ± 0.40	0.004
Total *n*-3	17.85 ± 0.44	0.79 ± 0.10	7.06 ± 0.17	6.31 ± 0.61	0.162
*n*-6:*n*-3 ratio	1.00 ± 0.03	1.01 ± 0.11	7.25 ± 0.31	8.96 ± 0.88	0.065

^1^ Means ± standard deviation (*n* = 3 per group). ^2^ Results of independent samples *t*-test between PBB feed and PCS feed. PBB feed, custom feed of peas, barley, alfalfa, and calcium; PCS feed, standard corn, and soy feed; <LLOD, below lower limit of detection; SFA, saturated fatty acids; MUFA, monounsaturated fatty acids, PUFA, polyunsaturated fatty acids.

**Table 3:** Physical characteristics of the eggs ^1^.

Parameters	PBB Eggs	PCS Eggs	CFC Eggs	*p*-Value ^2^
Egg weight (g)	56.98 ± 5.11	63.04 ± 8.49	64.27 ± 1.88	0.100
Yolk weight (g)	16.36 ± 1.78	17.94 ± 0.95	17.30 ± 1.45	0.191
Shell weight (g)	5.48 ± 0.77	5.70 ± 0.93	5.98 ± 0.40	0.515
Albumen weight (g)	35.15 ± 4.33	39.40 ± 8.50	40.99 ± 1.10	0.202
Haugh units	73.05 ± 6.33	81.51 ± 4.29	73.81 ± 7.39	0.057
Yolk color fan	14.00 ± 0.00	>14 ^3^	8.17 ± 0.98	<0.001 ^4^

^1^ Means ± standard deviation (*n* = 6 per group). ^2^ Results of one-way ANOVA. ^3^ All PCS egg yolks were darker orange than the color scale used. ^4^ Comparison between PBB and CFC using an independent samples *t*-test. PBB eggs, pasture-raised eggs with beef by-products and corn/soy free feed; PCS eggs, pasture-raised eggs with corn/soy feed; CFC eggs, cage-free comparison eggs.

**Table 4:** Antioxidant profile of the egg yolks ^1^.

Parameters	PBB Eggs	PCS Eggs	CFC Eggs	*p*-Value ^2^
Total carotenoids (µg/g fresh yolk)	41.54 ± 7.07 a	48.99 ± 12.35 a	18.23 ± 3.23 b	<0.001
Total phenols (mg of GAE/g fresh yolk)	0.60 ± 0.51 b	1.67 ± 0.82 a	1.80 ± 0.49 a	0.008

^1^ Means ± standard deviation (*n* = 6 per group). ^2^ Results of one-way ANOVA. a, b Means within a row with different letters significantly differ (*p* < 0.05). PBB eggs, pasture-raised eggs with beef by-products and corn/soy free feed; PCS eggs, pasture-raised eggs with corn/soy feed; CFC eggs, cage-free comparison eggs, GAE, gallic acid equivalents.

**Table 5:** Fatty acid profile of the egg yolks (% of total fatty acids) ^1^.

Fatty Acid	PBB Eggs	PCS Eggs	CFC Eggs	*p*-Value ^2^
10:0	0.02 ± 0.00	0.02 ± 0.01	0.02 ± 0.00	0.482
12:0	0.01 ± 0.00	0.01 ± 0.00	0.01 ± 0.00	0.442
14:0	0.56 ± 0.13 a	0.29 ± 0.10 b	0.27 ± 0.08 b	<0.001
14:1	0.13 ± 0.05 a	0.03 ± 0.01 b	0.02 ± 0.01 b	<0.001
16:0	26.60 ± 1.90	28.12 ± 1.80	28.53 ± 1.64	0.177
16:1 *n*-7	3.24 ± 0.88 a	2.54 ± 0.63 ab	1.71 ± 0.44 b	0.005
16:1 *n*-7 *t*	0.13 ± 0.04 a	0.09 ± 0.03 b	0.08 ± 0.01 b	0.004
16:1 *n*-9	0.70 ± 0.16 a	0.51 ± 0.09 b	0.37 ± 0.07 b	0.001
18:0	7.43 ± 0.37	7.00 ± 1.33	7.83 ± 1.45	0.479
18:1 *n*-7	1.97 ± 0.29 a	1.74 ± 0.21 a	1.14 ± 0.15 b	<0.001
18:1 *n*-9	46.62 ± 2.16 a	39.02 ± 2.74 b	35.18 ± 3.34 b	<0.001
18:1 *n*-9 *t*	0.32 ± 0.04 a	0.19 ± 0.04 b	0.17 ± 0.01 b	<0.001
18:2 *n*-6	7.08 ± 1.46 c	16.19 ± 3.91 b	21.76 ± 4.63 a	<0.001
18:3 *n*-3	0.40 ± 0.10 ab	0.62 ± 0.27 a	0.16 ± 0.04 b	0.001
18:3 *n*-6	0.11 ± 0.01 b	0.15 ± 0.05 ab	0.17 ± 0.01 a	0.015
20:0	0.04 ± 0.00	0.04 ± 0.02	0.04 ± 0.00	0.882
20:1 *n*-9	0.34 ± 0.06 a	0.19 ± 0.07 b	0.18 ± 0.02 b	<0.001
20:2 *n*-6	0.07 ± 0.04 b	0.19 ± 0.05 a	0.25 ± 0.08 a	<0.001
20:3 *n*-6	0.13 ± 0.01	0.16 ± 0.06	0.17 ± 0.01	0.171
20:4 *n*-6	0.61 ± 0.14	0.65 ± 0.13	0.79 ± 0.07	0.050
20:5 *n*-3	<LLOD	<LLOD	<LLOD	-
22:0	<LLOD	<LLOD	<LLOD	-
22:4 *n*-6	0.30 ± 0.10	0.33 ± 0.21	0.16 ± 0.14	0.176
22:5 *n*-3	0.33 ± 0.04 a	0.19 ± 0.12 b	<LLOD c	<0.001
22:5 *n*-6	0.26 ± 0.05 b	0.27 ± 0.08 b	0.38 ± 0.03 a	0.006
22:6 *n*-3	0.77 ± 0.10 a	0.93 ± 0.23 a	0.30 ± 0.05 b	<0.001
24:0	<LLOD	<LLOD	<LLOD	-
Total SFA	35.21 ± 2.10	35.74 ± 2.27	36.88 ± 2.27	0.431
Total MUFA	53.84 ± 1.66 a	44.42 ± 2.82 b	38.92 ± 3.68 c	<0.001
Total PUFA	10.36 ± 1.65 b	19.74 ± 3.72 a	24.19 ± 4.66 a	<0.001
Total *n*-6	8.55 ± 1.62 c	17.94 ± 3.68 b	23.67 ± 4.59 a	<0.001
Total *n*-3	1.50 ± 0.12 a	1.74 ± 0.39 a	0.47 ± 0.08 b	<0.001
*n*-6:*n*-3 ratio	5.72 ± 1.12 c	10.79 ± 3.30 b	50.63 ± 4.21 a	<0.001

^1^ Means ± standard deviation (*n* = 6 per group). ^2^ Results of one-way ANOVA. a–c Means within a row with different letters significantly differ (*p* < 0.05). PBB eggs, pasture-raised eggs with beef by-products and corn/soy free feed; PCS eggs, pasture-raised eggs with corn/soy feed; CFC eggs, cage-free comparison eggs; <LLOD, below lower limit of detection; SFA, saturated fatty acids; MUFA, monounsaturated fatty acids, PUFA, polyunsaturated fatty acids.

**Table 6:** Conjugated linoleic acid, odd-chain fatty acid, and branched-chain fatty acid content of grass-fed beef by-products, layer hen feeds, and egg yolks (% of total fatty acids) ^1^.

Fatty Acid	Beef Liver	Beef Suet	PBB Feed	PCS Feed	*p*-Value ^2^	PBB Eggs	PCS Eggs	CFC Eggs	*p*-Value ^3^
Conjugated linoleic acid									
9*c*, 11*t* 18:2	0.21 ± 0.03	0.26 ± 0.04	<LLOD	<LLOD	-	0.30 ± 0.13 a	0.06 ± 0.02 b	0.05 ± 0.00 b	<0.001
Odd-chain									
13:0	0.010 ± 0.003	0.021 ± 0.002	0.010 ± 0.002	0.004 ± 0.001	0.012	0.004 ± 0.001	0.004 ± 0.002	0.004 ± 0.000	0.541
15:0	0.29 ± 0.04	0.87 ± 0.11	0.11 ± 0.00	0.02 ± 0.00	<0.001	0.15 ± 0.04 a	0.06 ± 0.02 b	0.05 ± 0.01 b	<0.001
17:0	1.19 ± 0.05	1.10 ± 0.09	0.15 ± 0.01	0.09 ± 0.00	0.009	0.40 ± 0.11 a	0.18 ± 0.05 b	0.13 ± 0.02 b	<0.001
17:1	0.25 ± 0.05	0.40 ± 0.04	<LLOD	<LLOD	-	0.39 ± 0.13 a	0.13 ± 0.04 b	0.08 ± 0.01 b	<0.001
Total OCFA	1.74 ± 0.12	2.40 ± 0.24	0.26 ± 0.02	0.12 ± 0.01	0.001	0.95 ± 0.27 a	0.36 ± 0.12 b	0.26 ± 0.03 b	<0.001
Branched-chain									
15:0-*iso*	0.10 ± 0.01	0.42 ± 0.04	<LLOD	<LLOD	-	0.06 ± 0.03 a	0.02 ± 0.01 b	0.002 ± 0.005 b	<0.001
15:0-*anteiso*	0.08 ± 0.01	0.42 ± 0.04	<LLOD	<LLOD	-	0.02 ± 0.01 a	0.01 ± 0.01 b	0.004 ± 0.006 b	0.003
16:0-*iso*	0.08 ± 0.01	0.24 ± 0.03	<LLOD	<LLOD	-	0.05 ± 0.02 a	0.01 ± 0.00 b	<LLOD b	<0.001
17:0-*iso*	0.23 ± 0.00	0.44 ± 0.05	<LLOD	<LLOD	-	0.12 ± 0.04 a	0.02 ± 0.02 b	<LLOD b	<0.001
17:0-*anteiso*	0.37 ± 0.01	0.58 ± 0.05	<LLOD	<LLOD	-	0.29 ± 0.11 a	0.03 ± 0.01 b	0.002 ± 0.006 b	<0.001
18:0-*iso*	0.07 ± 0.01	0.09 ± 0.01	<LLOD	<LLOD	-	0.04 ± 0.02 a	<LLOD b	<LLOD b	<0.001
Total *iso*-BCFA	0.47 ± 0.02	1.20 ± 0.13	<LLOD	<LLOD	-	0.27 ± 0.10 a	0.06 ± 0.03 b	0.002 ± 0.005 b	<0.001
Total *anteiso*-BCFA	0.44 ± 0.01	1.00 ± 0.09	<LLOD	<LLOD	-	0.32 ± 0.12 a	0.04 ± 0.02 b	0.01 ± 0.01 b	<0.001
Total BCFA	0.91 ± 0.04	2.19 ± 0.22	<LLOD	<LLOD	-	0.59 ± 0.22 a	0.10 ± 0.05 b	0.008 ± 0.016 b	<0.001

^1^ Means ± standard deviation (*n* = 3 per group for feed, *n* = 6 per group for eggs). ^2^ Results of independent samples *t*-test between PBB feed and PCS feed. ^3^ Results of one-way ANOVA. a, b Means within a row with different letters significantly differ (*p* < 0.05). PBB feed, custom feed of peas, barley, alfalfa, and calcium; PCS feed, standard corn and soy feed; PBB eggs, pasture-raised eggs with beef by-products and corn/soy free feed; PCS eggs, pasture-raised eggs with corn/soy feed; CFC eggs, cage-free comparison eggs; <LLOD, below lower limit of detection; OCFA, odd-chain fatty acids; BCFA, branched-chain fatty acids.

**Table 7:** Vitamin profile of the egg yolks (μg/100 g fresh yolk) ^1^.

Vitamin	PBB Eggs	PCS Eggs	CFC Eggs	*p*-Value ^2^
Vitamin B1	296.32 ± 19.61 b	401.68 ± 38.89 a	234.88 ± 27.31 b	0.001
Vitamin B2	692.33 ± 109.72	705.05 ± 65.99	685.24 ± 93.17	0.965
Vitamin B3	16.64 ± 0.66 a	11.21 ± 1.76 b	10.06 ± 1.20 b	0.002
Vitamin B5	8077.25 ± 241.75 a	7260.07 ± 654.03 a	4190.95 ± 544.52 b	<0.001
Vitamin B9	0.15 ± 0.05	0.10 ± 0.01	0.18 ± 0.03	0.071
Vitamin B12	0.45 ± 0.06 b	0.49 ± 0.01 b	0.71 ± 0.03 a	<0.001
Vitamin A	246.24 ± 56.29	340.36 ± 24.07	221.94 ± 91.05	0.130
Vitamin D3	20.61 ± 2.94	21.75 ± 1.22	16.91 ± 5.58	0.318
Vitamin E ^3^	107.13 ± 60.77	93.16 ± 11.15	44.89 ± 19.30	0.182
Vitamin K1	0.53 ± 0.21 ab	0.97 ± 0.63 a	<LLOD b	0.055
Vitamin K2	0.57 ± 0.14	0.71 ± 0.15	0.87 ± 0.10	0.081

^1^ Means ± standard deviation (*n* = 3 per group). Each replicate was composed of two pooled egg yolks. ^2^ Results of one-way ANOVA. a, b Means within a row with different letters significantly differ (*p* < 0.05). ^3^ Vitamin E is reported as free alpha-tocopherol. PBB eggs, pasture-raised eggs with beef by-products and corn/soy free feed; PCS eggs, pasture-raised eggs with corn/soy feed; CFC eggs, cage-free comparison eggs; <LLOD, below lower limit of detection.

**Table 8:** Mineral profile of the egg yolks (mg/100 g fresh yolk) ^1^.

Mineral	PBB Eggs	PCS Eggs	CFC Eggs	*p*-Value ^2^
Sodium	36.70 ± 4.91	35.26 ± 6.02	45.86 ± 0.65	0.055
Magnesium	16.65 ± 1.42	17.02 ± 1.27	16.43 ± 0.74	0.830
Phosphorus	505.61 ± 45.77	502.22 ± 64.55	458.06 ± 13.74	0.428
Potassium	103.25 ± 12.71	90.64 ± 5.09	84.31 ± 2.70	0.070
Manganese	0.06 ± 0.01 b	0.11 ± 0.01 a	0.07 ± 0.00 b	<0.001
Iron	3.22 ± 0.45	2.53 ± 0.18	2.38 ± 0.39	0.060
Selenium	0.27 ± 0.02	0.33 ± 0.06	0.33 ± 0.12	0.635
Calcium	171.73 ± 18.43	161.87 ± 20.38	147.32 ± 5.29	0.254
Copper	0.13 ± 0.03	0.17 ± 0.07	0.13 ± 0.02	0.514
Zinc	3.00 ± 0.29	3.28 ± 0.47	3.94 ± 1.41	0.452

^1^ Means ± standard deviation (*n* = 3 per group). Each replicate was composed of two pooled egg yolks. ^2^ Results of one-way ANOVA. a, b Means within a row with different letters significantly differ (*p* < 0.05). PBB eggs, pasture-raised eggs with beef by-products and corn/soy free feed; PCS eggs, pasture-raised eggs with corn/soy feed; CFC eggs, cage-free comparison eggs.

## Data Availability

The data presented in this study are available in Data S1.

## Supplementary Materials

The following supporting information can be downloaded at: https://www.mdpi.com/article/10.3390/foods11213404/s1, Table S1: Fatty acid profile of grass-fed beef by-products and layer hen feeds (g fatty acid/100 g tissue or feed); Table S2: Fatty acid profile of the egg yolks (g fatty acid/100 g fresh yolk); Data S1: Data presented in this study.
