# Supplementary material for: Fatty Acid and Antioxidant Profile of Eggs from Pasture-Raised Hens Fed a Corn- and Soy-Free Diet and Supplemented with Grass-Fed Beef Suet and Liver
Source: Foods. 2022 Oct 28;11(21):3404. doi: 10.3390/foods11213404 (PMC9658713; doi:10.3390/foods11213404)
Supplement: Supplementary file 1 [file foods-11-03404-s001.zip › foods-1969225-supplementary/Supplementary Materials/Table S1.pdf]

Table S1. Fatty acid profile of grass-fed beef by-products and layer hen feeds (g fatty acid/100 g tissue or feed)[1]

Fatty acid	Beef liver	Beef suet	PBB feed	PCS feed	*P*-value[2]
10:0	<LLOD	0.02 ± 0.01	0.0004 ± 0.0000	0.0007 ± 0.0001	0.004
12:0	0.0002 ± 0.0000	0.03 ± 0.01	0.0001 ± 0.0000	0.0003 ± 0.0001	0.043
13:0	0.0002 ± 0.0000	0.007 ± 0.002	0.0001 ± 0.0000	0.0001 ± 0.0000	0.016
14:0	0.006 ± 0.001	1.37 ± 0.35	0.0024 ± 0.0004	0.0022 ± 0.0003	0.529
14:1	0.0004 ± 0.0001	0.10 ± 0.03	<LLOD	<LLOD	-
15:0	0.005 ± 0.001	0.31 ± 0.10	0.001 ± 0.000	0.001 ± 0.000	0.097
16:0	0.26 ± 0.08	9.29 ± 2.17	0.20 ± 0.03	0.46 ± 0.04	0.001
16:1 n-7	0.009 ± 0.003	0.58 ± 0.15	0.001 ± 0.000	0.003 ± 0.001	0.025
16:1 n-7 *t*	0.004 ± 0.001	0.04 ± 0.01	<LLOD	<LLOD	-
16:1 n-9	0.004 ± 0.001	0.13 ± 0.05	0.001 ± 0.000	0.002 ± 0.000	0.003
17:0	0.02 ± 0.01	0.39 ± 0.11	0.002 ± 0.000	0.004 ± 0.000	0.001
17:1	0.004 ± 0.001	0.14 ± 0.04	<LLOD	<LLOD	-
18:0	0.64 ± 0.22	8.37 ± 1.91	0.02 ± 0.00	0.10 ± 0.01	0.007
18:1 n-7	0.02 ± 0.01	0.34 ± 0.06	0.01 ± 0.00	0.04 ± 0.01	0.022
18:1 n-9	0.14 ± 0.04	10.67 ± 2.54	0.22 ± 0.04	0.81 ± 0.09	0.003
18:1 n-9 *t*	0.03 ± 0.01	1.92 ± 0.53	<LLOD	<LLOD	-
18:2 n-6	0.07 ± 0.02	0.28 ± 0.07	0.57 ± 0.10	2.17 ± 0.24	0.003
9*c*, 11*t* 18:2	0.004 ± 0.001	0.09 ± 0.03	<LLOD	<LLOD	-
18:3 n-3	0.04 ± 0.01	0.28 ± 0.10	0.08 ± 0.01	0.25 ± 0.05	0.025
18:3 n-6	0.004 ± 0.001	<LLOD	<LLOD	<LLOD	-
20:0	0.002 ± 0.000	0.05 ± 0.02	0.004 ± 0.000	0.008 ± 0.001	0.024
20:1 n-9	<LLOD	0.09 ± 0.02	0.005 ± 0.001	0.008 ± 0.001	0.016
20:2 n-6	0.004 ± 0.001	<LLOD	<LLOD	<LLOD	-
20:3 n-6	0.06 ± 0.02	<LLOD	<LLOD	<LLOD	-
20:4 n-6	0.13 ± 0.04	<LLOD	<LLOD	<LLOD	-
20:5 n-3	0.06 ± 0.02	<LLOD	<LLOD	<LLOD	-
22:0	<LLOD	0.02 ± 0.00	0.002 ± 0.000	0.007 ± 0.001	0.012
22:4 n-6	0.04 ± 0.02	<LLOD	<LLOD	<LLOD	-
22:5 n-3	0.18 ± 0.06	<LLOD	<LLOD	<LLOD	-
22:6 n-3	0.05 ± 0.02	<LLOD	<LLOD	<LLOD	-
24:0	<LLOD	<LLOD	0.004 ± 0.000	0.007 ± 0.001	0.022
15:0-*iso*	0.002 ± 0.000	0.15 ± 0.04	<LLOD	<LLOD	-
15:0-*anteiso*	0.001 ± 0.000	0.15 ± 0.04	<LLOD	<LLOD	-
16:0-*iso*	0.001 ± 0.000	0.09 ± 0.03	<LLOD	<LLOD	-
17:0-*iso*	0.004 ± 0.001	0.16 ± 0.05	<LLOD	<LLOD	-
17:0-*anteiso*	0.007 ± 0.002	0.21 ± 0.06	<LLOD	<LLOD	-
18:0-*iso*	0.001 ± 0.000	0.03 ± 0.01	<LLOD	<LLOD	-
Total SFA	0.93 ± 0.31	19.85 ± 4.65	0.24 ± 0.03	0.59 ± 0.05	0.001
Total MUFA	0.21 ± 0.06	14.02 ± 3.40	0.23 ± 0.04	0.86 ± 0.10	0.004
Total PUFA	0.64 ± 0.21	0.66 ± 0.21	0.65 ± 0.11	2.42 ± 0.29	0.004
Total n-6	0.31 ± 0.11	0.28 ± 0.07	0.57 ± 0.10	2.17 ± 0.24	0.003
Total n-3	0.32 ± 0.11	0.28 ± 0.10	0.08 ± 0.01	0.25 ± 0.05	0.025
n-6:n-3 ratio	0.98 ± 0.03	1.01 ± 0.11	7.24 ± 0.31	8.95 ± 0.88	0.065
Total OCFA	0.03 ± 0.01	0.85 ± 0.25	0.003 ± 0.000	0.005 ± 0.000	0.004
Total *iso*-BCFA	0.01 ± 0.00	0.42 ± 0.13	<LLOD	<LLOD	-
Total *anteiso*-BCFA	0.01 ± 0.00	0.35 ± 0.11	<LLOD	<LLOD	-
Total BCFA	0.02 ± 0.01	0.78 ± 0.23	<LLOD	<LLOD	-
Total FA	1.80 ± 0.58	35.30 ± 8.47	1.12 ± 0.18	3.87 ± 0.44	0.003

[1]Data are reported as means ± standard deviation (n = 3 per group). [2]*P*-values indicate results of independent samples t-test between PBB feed and PCS feed.

PBB feed, custom feed of peas, barley, alfalfa, and calcium; PCS feed, standard corn and soy feed; <LLOD, below lower limit of detection; SFA, saturated fatty acids; MUFA, monounsaturated fatty acids, PUFA, polyunsaturated fatty acids; OCFA, odd chain fatty acids; BCFA, branched chain fatty acids; FA, fatty acids
