# Supplementary material for: Fatty Acid and Antioxidant Profile of Eggs from Pasture-Raised Hens Fed a Corn- and Soy-Free Diet and Supplemented with Grass-Fed Beef Suet and Liver
Source: Foods. 2022 Oct 28;11(21):3404. doi: 10.3390/foods11213404 (PMC9658713; doi:10.3390/foods11213404)
Supplement: Supplementary file 1 [file foods-11-03404-s001.zip › foods-1969225-supplementary/Supplementary Materials/Table S2.pdf]

Table S2. Fatty acid profile of the egg yolks (g fatty acid/100 g fresh yolk)[1]

Fatty acid	PBB eggs	PCS eggs	CFC eggs	*P*-value[2]
10:0	0.003 ± 0.001	0.004 ± 0.001	0.003 ± 0.000	0.237
12:0	0.001 ± 0.000	0.001 ± 0.000	0.001 ± 0.000	0.752
13:0	0.0007 ± 0.0002	0.0007 ± 0.0002	0.0006 ± 0.0000	0.449
14:0	0.09 ± 0.04 a	0.05 ± 0.02 b	0.04 ± 0.02 b	0.012
14:1	0.02 ± 0.01 a	0.005 ± 0.002 b	0.003 ± 0.002 b	<0.001
15:0	0.02 ± 0.01 a	0.01 ± 0.00 b	0.007 ± 0.001 b	0.001
16:0	4.10 ± 1.12	4.62 ± 0.77	4.47 ± 0.83	0.615
16:1 n-7	0.51 ± 0.20 a	0.41 ± 0.11 ab	0.27 ± 0.09 b	0.028
16:1 n-7 *t*	0.02 ± 0.01 a	0.01 ± 0.01 ab	0.01 ± 0.00 b	0.034
16:1 n-9	0.10 ± 0.03 a	0.08 ± 0.02 ab	0.06 ± 0.01 b	0.002
17:0	0.06 ± 0.03 a	0.03 ± 0.01 b	0.02 ± 0.00 b	0.001
17:1	0.06 ± 0.03 a	0.02 ± 0.01 b	0.02 ± 0.00 b	<0.001
18:0	1.13 ± 0.26	1.17 ± 0.41	1.23 ± 0.30	0.888
18:1 n-7	0.30 ± 0.09 a	0.28 ± 0.05 a	0.18 ± 0.03 b	0.009
18:1 n-9	7.12 ± 1.67	6.43 ± 1.37	5.46 ± 0.67	0.121
18:1 n-9 *t*	0.05 ± 0.02 a	0.03 ± 0.01 b	0.03 ± 0.00 b	0.002
18:2 n-6	1.07 ± 0.26 b	2.61 ± 0.58 a	3.38 ± 0.78 a	<0.001
9*c*, 11*t* 18:2	0.05 ± 0.02 a	0.01 ± 0.00 b	0.01 ± 0.00 b	<0.001
18:3 n-3	0.06 ± 0.02 ab	0.10 ± 0.04 a	0.03 ± 0.01 b	0.001
18:3 n-6	0.02 ± 0.00 b	0.02 ± 0.01 a	0.03 ± 0.00 a	0.007
20:0	0.006 ± 0.001	0.007 ± 0.002	0.006 ± 0.001	0.699
20:1 n-9	0.05 ± 0.02 a	0.03 ± 0.01 b	0.03 ± 0.01 b	0.006
20:2 n-6	0.01 ± 0.01 b	0.03 ± 0.01 a	0.04 ± 0.01 a	0.001
20:3 n-6	0.02 ± 0.00	0.03 ± 0.01	0.03 ± 0.00	0.164
20:4 n-6	0.09 ± 0.02	0.11 ± 0.04	0.12 ± 0.03	0.195
20:5 n-3	<LLOD	<LLOD	<LLOD	-
22:0	<LLOD	<LLOD	<LLOD	-
22:4 n-6	0.04 ± 0.01	0.06 ± 0.04	0.03 ± 0.02	0.187
22:5 n-3	0.05 ± 0.01 a	0.03 ± 0.03 a	<LLOD b	<0.001
22:5 n-6	0.04 ± 0.01	0.05 ± 0.02	0.06 ± 0.01	0.058
22:6 n-3	0.12 ± 0.03 a	0.16 ± 0.06 a	0.05 ± 0.01 b	0.001
24:0	<LLOD	<LLOD	<LLOD	-
15:0-*iso*	0.01 ± 0.01 a	0.003 ± 0.001 b	0.0004 ± 0.0009 b	0.001
15:0-*anteiso*	0.004 ± 0.002 a	0.002 ± 0.001 ab	0.0006 ± 0.0009 b	0.011
16:0-*iso*	0.008 ± 0.004 a	0.002 ± 0.001 b	<LLOD b	<0.001
17:0-*iso*	0.02 ± 0.01 a	0.004 ± 0.003 b	<LLOD b	<0.001
17:0-*anteiso*	0.05 ± 0.02 a	0.006 ± 0.002 b	0.0004 ± 0.0009 b	<0.001
18:0-*iso*	0.007 ± 0.003 a	<LLOD b	<LLOD b	<0.001
Total SFA	5.42 ± 1.44	5.89 ± 1.15	5.78 ± 1.05	0.787
Total MUFA	8.25 ± 2.00	7.31 ± 1.48	6.05 ± 0.77	0.067
Total PUFA	1.56 ± 0.34 b	3.20 ± 0.58 a	3.76 ± 0.82 a	<0.001
Total n-6	1.29 ± 0.30 b	2.90 ± 0.57 a	3.68 ± 0.80 a	<0.001
Total n-3	0.23 ± 0.06 a	0.29 ± 0.09 a	0.07 ± 0.02 b	<0.001
n-6:n-3 ratio	5.72 ± 1.12 c	10.79 ± 3.30 b	50.63 ± 4.21 a	<0.001
Total OCFA	0.15 ± 0.06 a	0.06 ± 0.01 b	0.04 ± 0.00 b	<0.001
Total *iso*-BCFA	0.04 ± 0.02 a	0.009 ± 0.004 b	0.0004 ± 0.0009 b	<0.001
Total *anteiso*-BCFA	0.05 ± 0.03 a	0.007 ± 0.003 b	0.001 ± 0.002 b	<0.001
Total BCFA	0.09 ± 0.05 a	0.02 ± 0.01 b	0.001 ± 0.003 b	<0.001
Total FA	15.32 ± 3.64	16.42 ± 2.67	15.59 ± 2.06	0.790

[1]Data are reported as means ± standard deviation (n = 6 per group). [2]*P*-values indicate results of one-way ANOVA. Means within a row that have different letters are significantly different according to Tukey's HSD test (*P*<0.05). PBB eggs, pasture-raised eggs with beef by-products and corn/soy free feed; PCS eggs, pasture-raised eggs with corn/soy feed; CFC eggs, cage-free comparison eggs; <LLOD, below lower limit of detection; SFA, saturated fatty acids; MUFA, monounsaturated fatty acids, PUFA, polyunsaturated fatty acids; OCFA, odd chain fatty acids; BCFA, branched chain fatty acids; FA, fatty acids
